# Concept of natural genome reconstruction. Part 3. Analysis of changes in the amount of telomeric DNA in colony cells as a new amplified feature that arose during the processing of hematopoietic bone marrow stem cells

**DOI:** 10.18699/vjgb-25-52

**Published:** 2025-07

**Authors:** V.S. Ruzanova, S.G. Oshikhmina, G.S. Ritter, E.V. Dolgova, S.S. Kirikovich, E.V. Levites, Y.R. Efremov, T.V. Karamysheva, A.G. Bogomolov, M.I. Meschaninova, A.L. Mamaev, O.S. Taranov, S.V. Sidorov, S.D. Nikonov, O.Y. Leplina, A.A. Ostanin, E.R. Chernykh, N.A. Kolchanov, A.S. Proskurina, S.S. Bogachev

**Affiliations:** Institute of Cytology and Genetics of the Siberian Branch of the Russian Academy of Sciences, Novosibirsk, Russia; Institute of Cytology and Genetics of the Siberian Branch of the Russian Academy of Sciences, Novosibirsk, Russia Novosibirsk State University, Novosibirsk, Russia; Institute of Cytology and Genetics of the Siberian Branch of the Russian Academy of Sciences, Novosibirsk, Russia; Institute of Cytology and Genetics of the Siberian Branch of the Russian Academy of Sciences, Novosibirsk, Russia; Institute of Cytology and Genetics of the Siberian Branch of the Russian Academy of Sciences, Novosibirsk, Russia; Institute of Cytology and Genetics of the Siberian Branch of the Russian Academy of Sciences, Novosibirsk, Russia; Institute of Cytology and Genetics of the Siberian Branch of the Russian Academy of Sciences, Novosibirsk, Russia; Institute of Cytology and Genetics of the Siberian Branch of the Russian Academy of Sciences, Novosibirsk, Russia; Institute of Cytology and Genetics of the Siberian Branch of the Russian Academy of Sciences, Novosibirsk, Russia; Institute of Chemical Biology and Fundamental Medicine of the Siberian Branch of the Russian Academy of Sciences, Novosibirsk, Russia; Laboratory Angiopharm LLC, Novosibirsk, Russia; State Scientific Center of Virology and Biotechnology “Vector” of Rospotrebnadzor, Koltsovo, Novosibirsk region, Russia; Novosibirsk State University, Novosibirsk, Russia City Clinical Hospital No. 1, Novosibirsk, Russia; Novosibirsk Tuberculosis Research Institute, Novosibirsk, Russia; Research Institute of Fundamental and Clinical Immunology, Novosibirsk, Russia; Research Institute of Fundamental and Clinical Immunology, Novosibirsk, Russia; Research Institute of Fundamental and Clinical Immunology, Novosibirsk, Russia; Institute of Cytology and Genetics of the Siberian Branch of the Russian Academy of Sciences, Novosibirsk, Russia; Institute of Cytology and Genetics of the Siberian Branch of the Russian Academy of Sciences, Novosibirsk, Russia; Institute of Cytology and Genetics of the Siberian Branch of the Russian Academy of Sciences, Novosibirsk, Russia

**Keywords:** hematopoietic stem cells, dot blot hybridization, telomeric DNA, angiogenin, recombinogenic situation, гемопоэтические стволовые клетки, дот-блот гибридизация, теломерная ДНК, ангиогенин, рекомбиногенная ситуация

## Abstract

The induced “recombinogenic situation” in hematopoietic stem cells and the activation of the cell’s reparative systems create the basis for recombination events between fragments of extracellular double-stranded DNA delivered into the cell and chromosomal DNA or other forms of the reparative-recombination process. In mouse and rat model organisms as well as in human bone marrow cells, changes in the amount of telomeric DNA in hematopoietic stem cells were assessed as an indicator of repair and recombination events that have occurred. In all experiments performed, recombinant human angiogenin was used as a comparison factor. Dot blot hybridization showed that in the colony cells obtained from the bone marrow cells of the model organisms as well as from human bone marrow cells treated with a double-stranded DNA preparation, there was a significant increase in the amount of telomeric DNA. Amplification of telomeric DNA in colony cells is not associated with contamination of the original DNA preparation with which the bone marrow cells were treated. Treatment of bone marrow cells with DNA that does not carry telomeric sequences (AluI PCR fragment) does not lead to an increase in the amount of telomeric DNA in the cells of grown colonies. This suggests the participation in the amplification of telomeric DNA of an extrachromosomal DNA template carrying telomeric DNA. It has been established that treatment of bone marrow cells with angiogenin also leads to an increase in telomeric DNA in colony cells. A comparison of the type of colonies with the intensity of hybridization (i. e. the amount of telomeric DNA in the sample) suggests that the increase in the amount of detectable telomeric DNA following treatment with angiogenin and hDNAgr has a fundamentally different origin. Western blot analysis and real-time PCR revealed that the increase in the amount of telomeric DNA following treatment of bone marrow cells with a double-stranded DNA preparation does not correlate with the activity of endogenous/exogenous telomerase. For angiogenin, it has been shown that an increase in the amount of telomeric DNA may be the result of activation of endogenous telomerase activity. A principle has been developed for the amplification of a new genetic trait that came into hematopoietic stem cells with extracellular double-stranded DNA material and was fixed in the recipient genome or was transitively present in the cell as new genetic information.

## Abstract (ru)

Индуцированная «рекомбиногенная ситуация» в гемопоэтических стволовых клетках и активация репаративных систем клетки создают основу для рекомбинационных событий между доставленными в клетку фрагментами экстраклеточной двуцепочечной ДНК и ДНК хромосом или иных форм репаративно-рекомбинационного процесса. На модельных организмах мыши и крысы, а также с использованием в качестве исходного материала клеток костного мозга человека было оценено изменение количества теломерной ДНК в гемопоэтических стволовых клетках как показатель произошедших репарационно-рекомбинационных событий. Во всех проведенных экспериментах в качестве фактора сравнения использовался ангиогенин рекомбинантный человеческий. Методом дот-блот гибридизации показано, что в клетках колоний, полученных из клеток костного мозга модельных организмов, а также из клеток образцов костного мозга человека, обработанных препаратом двуцепочечной ДНК, произошло достоверное увеличение количества теломерной ДНК. Амплификация теломерной ДНК в клетках колоний не связана с контаминацией препаратом исходной ДНК, которым обрабатывались клетки костного мозга. Обработка клеток костного мозга ДНК, не несущей теломерных последовательностей (AluI ПЦР-фрагмент), не приводит к увеличению количества теломерной ДНК в клетках выросших колоний. Это предполагает участие в амплификации теломерной ДНК экстрахромосомальной ДНК-матрицы, несущей ДНК теломер. Установлено, что обработка клеток костного мозга ангиогенином также сопровождается увеличением теломерной ДНК в клетках колоний. Сопоставление типа колоний с интенсивностью гибридизации (т. е. количества теломерной ДНК в образце) предполагало, что увеличение количества детектируемой теломерной ДНК при обработке ангиогенином и hDNAgr имеет принципиально разное происхождение. Вестерн-блот анализом и методом ПЦР в реальном времени установлено, что увеличение количества теломерной ДНК при обработке клеток костного мозга препаратом двуцепочечной ДНК не коррелирует с активностью эндогенной/экзогенной теломеразы. Для ангиогенина показано, что увеличение количества теломерной ДНК может быть результатом активации эндогенной теломеразной активности. Разработан принцип амплификации нового генетического признака, пришедшего в гемопоэтические стволовые клетки с экстраклеточным двуцепочечным ДНК материалом и закрепившимся в реципиентном геноме или транзитно присутствующим в клетке в качестве новой генетической информации.

## Introduction

The central idea of this part of the study is to prove that extracellular double-stranded DNA fragments internalized into hematopoietic stem cells (HSCs) (Ruzanova et al., 2024) are involved in recombination repair processes activated by these fragments in undifferentiated precursors. A telomere, which consists of repetitive homogeneous DNA sequences, was used as the model target as changes in its content can be easily detected experimentally. In all the mammals, telomeric repeats have an identical nucleotide sequence. Therefore, human DNA can be used as a substrate for assessing changes in telomeric DNA content caused by recombination repair processes in different experimental model systems. Quantitative dot blot hybridization with a telomeric repeat DNA probe was chosen as the main method for assessing the events that have taken place.

Induction of pangenomic single-strand breaks by doublestranded DNA fragments internalized into HSCs via a natural mechanism is the underlying phenomenon in a cascade of events defined by us as a “recombinogenic situation” (Likhacheva et al., 2008). This state of the cell drives the recombination repair machinery, resulting in numerous interactions between chromatin and intranuclear DNA fragments.

In essence, it is the same situation as the one when doublestrand breaks are formed or disruption of higher-order chromatin structure is induced in the cell. Two key aspects of this process can be differentiated within the recombinogenic situation: the enzymatic molecular machinery activated in the cell and recombinant intermediates of chromatin and DNA fragments involved in recombination repair. Both aspects have been thoroughly analyzed for double-strand breaks and single-stranded DNA, while the data on nick-initiated recombinogenic situation are very sparse as there has been a lack of research community’s attention to these processes over the past two decades.

According to what has been said, in Supplementary Materials 1 and 21, we briefly describe the molecular events characterizing the emergence of double-strand breaks and single-stranded DNA or disruption of higher-order chromatin structure assuming that many of the described details will also be typical of the nick-initiated recombinogenic situation. Supplementary Material 1 lists brief information about factors involved in the processes described. An analysis reported in Supplementary Material 2 indicates that a comprehensive response to damage and various perturbations in higher-order chromatin structure is induced in the cell. The system of hierarchical kinases (ATM, ATR, DNA-PK, belonging to the family of phosphatidylinositol-3-kinase-dependent kinases) is activated, and molecular systems of either restoring chromatin integrity or normalizing its spatial organization are brought into an active state.


Supplementary Materials are available in the online version of the paper: https://vavilov.elpub.ru/jour/manager/files/Suppl_Ruzanova_Engl_29_4.pdf


Single-strand DNA breaks (nicks) are among the key factors initiating the disruption of higher-order chromatin structure. This type of chromatin structure disruption was shown to have its own repair pathway and activate the palette of recombination repair factors that is intrinsic to this pathway and differs from the machinery of double-strand DNA break repair. Homologous recombination, the main feature of which is the high precision of correction of genetic information, is activated upon nick-induced recombinogenic situation (Vriend, Krawczyk, 2017; Maizels, Davis, 2018). It is known that ATM and ATR kinases are not absolutely required for the DNA break repair process upon nick emergence and repair via the homologous recombination mechanism. It means that nick-initiated homologous recombination may proceed without involvement of hierarchically organized kinases and, therefore, without activation of the checkpoint mechanism. Furthermore, there are grounds to believe that nick-initiated homologous recombination can be independent of the phase of the cell cycle. Like double-strand break repair, repair of DNA nicks depends on the formation of replication factor A filaments. While double-strand break repair is dependent on activity of the BRCA1, RAD51, and BRCA2 complexes, repair of DNA nicks is related to BRCA1 activity, but is independent of RAD51 (Vriend, Krawczyk, 2017; Maizels, Davis, 2018).

Therefore, DNA fragments internalized into the cell induce nicks. A recombinogenic situation develops, and the homologous recombination mechanism is initiated. In this scenario, intracellular DNA fragments act as an extrachromosomal substrate for recombination repair processes activated by them. As a result of the interaction of fragments and chromatin, changes in the DNA of chromosomes will occur. We suppose that if these alterations are large-scale, they can be detected by modern analytical methods.

Chromosomal loci in which genomic alterations can be detected will obviously be those carrying repetitive DNA sequences, including intercalary heterochromatin domains, centromeres, and telomeres. The DNA content in these chromosome domains is high, so changes in DNA content in a selected locus can be detected using various experimental approaches, including real-time PCR, fluorescent in situ hybridization (FISH), and quantitative dot blot hybridization assay, in the case of large-scale alterations. Intercalary heterochromatin and centromeric satellites are species-specific, and allogeneic DNA should be used for analyzing alterations that have occurred in the genome. In all mammals and humans, telomeric satellites are represented by the same hexanucleotide repeat TTAGGG, and any mammalian or human doublestranded DNA can be used for conducting experiments and analyzing telomeric chromatin alterations in different species (Giardini et al., 2014).

**Fig. 1.**
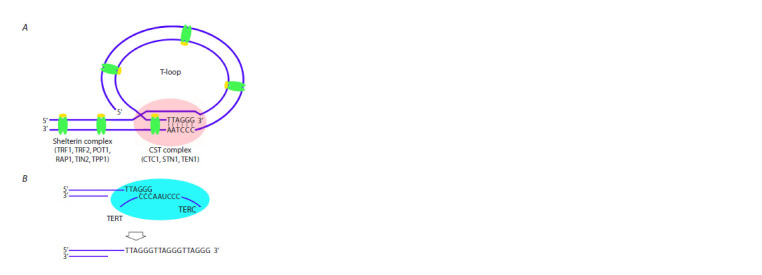
Telomere structure and the mechanism of its elongation by the telomerase complex А – protein complexes in the telomeric region. B – the mechanism of telomere lengthening by the telomerase complex. Several nucleotides at the 3’ end of the telomeric G-strand complementarily bind to the TERC template sequence of telomerase RNA. The chromosome end is lengthened by telomerase reverse transcriptase (TERT).

Telomeres are specific chromatin structures at the ends of eukaryotic chromosomes. As mentioned above, telomeric DNA in most eukaryotes consists of hexanucleotide repeats (for humans, TTAGGG). Human telomeres are approximately 10 kbp long. The forward and complementary telomeric strands are known as the G-strand and the C-strand, respectively. The 3ʹ-end of the G-strand is single-stranded DNA known as the telomeric G-tail. It has been demonstrated that the G-tail penetrates into the proximal double-stranded repeat and anneals to the C-strand to form a special structure, the t-loop. Each telomere is the region of DNA sequence at the end of a chromosome that is protected by the closed circle of the t-loop structure and specific proteins, shelterin and CST (CTC1-STN1-TEN1) heterotrimeric complex, against degradation causing chromosomal instability. The CST heterotrimer binds to the t-loop intermediate at the site of annealing of the single-stranded G-overhang and the complementary sequence of the 3ʹ–5ʹ strand (C-strand) to form a protective capping complex (Fig. 1A) (Giardini et al., 2014; Soman et al., 2022; Alanazi et al., 2024).

Shelterin, a specialized protein complex, is a functional basis of telomeric chromatin and, in mammalian cells, consists of one (POT1) and two (TRF1 and TRF2) telomeric DNA binding proteins, as well as specific proteins linking these DNA binding proteins (Fig. 1A) (Giraud-Panis et al., 2010; Lee et al., 2014; Soman et al., 2022). Together, these structural complexes form telomeric heterochromatin in mammals (Lu W. et al., 2013).

Telomeric DNA in dividing cells is prone to shortening (the so-called end replication problem). Semiconservative replication cannot complete the synthesis of ends of linear DNA. Hence, after several cell division rounds, somatic cells have shortened telomeric DNA, resulting in irreversible cell cycle arrest (the so-called replicative senescence) (Chan, Blackburn, 2003; Doksani, 2019; Jones et al., 2023).

Two mechanisms preventing telomere shortening and preserving the feasibility of infinite division have been described; these mechanisms are active in stem cells, including HSCs, and in immortalized cancer cells. The main mechanism is related to telomerase activity (Fig. 1B). Telomerase is a specific reverse transcriptase that elongates the telomeric G-strand. Stem cells and cancer cells in ~90 % of tumors maintain telomere length in a telomerase-dependent manner (Chan, Blackburn, 2003; Nandakumar, Cech, 2013).

The second mechanism, known as alternative lengthening of telomere, has been described for a small number of tumors. This pathway is characterized by a specific mechanism of telomeric DNA metabolism where the key elements are recombination and replication-associated recombination (Lundblad, 2002; Hande, 2004; Pickett et al., 2009; Nabetani, Ishikawa, 2011; Rovatsos et al., 2011; Doksani, 2019; Loe et al., 2020; Lu R., Pickett, 2022; Jones et al., 2023).

In the present study, for assessing large-scale genomic alterations, we chose to analyze the content of telomeric DNA (as a target consisting of non-species-specific repeats in mammals) in cells treated with therapeutic DNA, hDNAgr. The analysis was performed using three approaches: FISH, real-time PCR, and dot blot hybridization assay (Supplementary Material 3). As mentioned above, a simple way to assess changes in the telomeric DNA content in HSCs will be to analyze this parameter in progeny cells after treatment of HSCs within bone marrow cells and their amplification to form colonies on methylcellulose (up to 1,000 cells per colony). Genetically altered HSCs on methylcellulose will produce genetically homogeneous progeny. In other words, a new detectable trait will be amplified (the technology is the property of OJSC “ES.LAB DIAGNOSTIC”, Patent Application No. 2023124343 dated September 20, 2023). Telomeres are formed by repeats that are identical for all mammals (the TTAGGG repeat sequence in vertebrates/humans). In principle, this fact made it possible to use hDNAgr in the mouse or rat models to assess alterations in the telomeric DNA content.

Additionally, changes in the telomerase level in colony cells were assessed. Fifteen days after the initial induction within bone marrow cells, colony cells were re-treated with the same factors. Evaluation was performed for colony cell samples collected at time points 0 (untreated samples) and 1, 2, 4, 8, 16, and 32 h after re-treatment. All the evaluations involved comparison between cells treated with three inducers: angiogenin, hDNAgr, and angiogenin+hDNAgr.

## Materials and methods

Experimental animals. Young male CBA/Lac mice aged 2–5 months, old male CBA/Lac mice aged 9–12 months, and old male Wistar rats aged 18–22 months, bred at the Conventional Vivarium (Institute of Cytology and Genetics, SB RAS; Novosibirsk, Russia), were used in this study. The animals were housed in groups (6–10 mice and 3–4 rats per cage) with ad libitum access to food and water. All animal experiments were approved by the Animal Care and Use Committee of the Institute of Cytology and Genetics, SB RAS. Mice were withdrawn from the experiment by cervical dislocation; rats, by euthanasia using CO2 or decapitation.

Human bone marrow cells. Cells from cryopreserved bone marrow specimens collected from patients with Hodgkin lymphoma, provided by the cryobank of the Research Institute of Fundamental and Clinical Immunology, were utilized. At the Clinic of Immunopathology of the Research Institute of Fundamental and Clinical Immunology (Hematology Department), having a bone marrow transplant unit, patients with hemoblastosis receive high-dose chemotherapy and transplantation of autologous or allogeneic peripheral HSCs. When harvesting peripheral stem cells, along with the main apheresis product (which is transplanted to the patient), two or three samples (satellite test tubes) of separated cells are also collected to ensure quality control of the apheresis product and for research purposes. Together with the main specimen, these samples were used in the present study. Each bone marrow specimen, including satellite ones, is accompanied by the required documentation package including the Informed Consent Form, Protocol of Bone Marrow Examination, and Treatment Protocol, which are signed by the patient in accordance with the statutory standards. After the treatment and application of the main product, the satellite samples are either disposed of in compliance with the Sanitary Rules and Regulations or used for research purposes. Documents accompanying each procedure of bone marrow harvesting are stored in the archive of the cryobank of the Research Institute of Fundamental and Clinical Immunology and can be claimed upon first demand.

DNA preparation. Human DNA genome reconstructor (hDNAgr) and placenta DNA were isolated from placentas of healthy women. hDNAgr was fragmented to 1–10 nucleosome monomers (200–2,000 bp) by ultrasonic disintegration, deproteinized using proteinase K, and isolated by phenol–chloroform extraction. Placenta DNA was extracted in a similar manner, without fragmentation.

Angiogenin was procured from the Angiopharm Laboratory LLC (Novosibirsk, Russia)

pBSM13-AluI-pBSM13 PCR fragment. The human AluI repeat (the pBSM13-AluI-pBSM13 fragment) was amplified by PCR. AluI repeat DNA cloned into pUC19, including the beginning and end of the tandemly repeated AluJ and AluY sequences (NCBI: AC002400.1, 53494–53767), was used as a template. Standard M13 primers were used for amplification (M13 for: 5ʹ GTAAAACGACGGCCAGT 3ʹ; M13 rev: 5ʹ CAGGAAACAGCTATGAC 3ʹ). The PCR fragment was resuspended in 0.1 V NaAc 3 M pH 5.2 and 1 V isopropanol for 10 min at –20 °C. The precipitate was washed in 70 % ethanol and dissolved in sterile water.

Isolation of bone marrow cells. After cervical dislocation, femoral and tibial bones were isolated, epiphyses were removed, and the bone marrow cavity was washed with IMDM+2 % FBS. The resulting cell suspension was passed through a 21-gauge needle several times to eliminate bone marrow rosettes and then through a 40-μm filter. Cells were pelleted by centrifuging for 10 min at 400g and resuspended in red blood cell lysis buffer containing 130 mM ammonium chloride for 3–5 min. The buffer was then diluted tenfold with PBS; cells were re-pelleted, resuspended in IMDM medium, and counted in a Goryaev chamber.

Treatment of bone marrow cells with inducers. Bone marrow cells isolated from old animals and bone marrow sections from patients with Hodgkin lymphoma were incubated with inducers for 1 h in the 5 % CO2 atmosphere with 95 % humidity at 37 ℃ at the following ratio: 500 μg of hDNAgr, or 500 ng of angiogenin, or 500 μg of hDNAgr + 500 ng angiogenin in 1 mL of serum-free IMDM medium per 3×106 cells. Control (untreated) bone marrow cells were incubated in serum-free IMDM medium supplemented with PBS volume equal to that of the inducer added to activate bone marrow cells.

Cultivation of bone marrow cells in methylcellulose medium. Bone marrow cells with/without inducer activation were pelleted for 10 min at 400g and resuspended in IMDM+2 % FBS. To quantify and analyze myeloid precursors, the mouse bone marrow cells were placed in the MethoCult M3434 methylcellulose medium; the rat and human bone marrow cells were placed in the MethoCult H4034 methylcellulose medium (Stem Cell Technologies). Colony counting and cell isolation from the methylcellulose medium after cultivation were carried out according to the manufacturer’s instructions. Cells were cultivated for 9–15 days depending on the experiment objective.

DNA isolation from colony cells and the liver of young mice. Colony cells were pelleted at 400g for 5–7 min; the precipitate was resuspended in 50 mM EDTA.

After mice had been subjected to cervical dislocation, a liver fragment was dissected and homogenized in a buffer supplemented with 100 mM EDTA pH 8.0 and 20 mM Tris- HCl pH 7.5. SDS was then added to the cells in both cases until a concentration of 1 %, and the homogenate was incubated in the presence of 100 μg/mL proteinase K at 58 °C for 60 min. DNA was isolated by phenol-chloroform extraction and repelleting of 1 V isopropanol from 0.3 M NaAc. The pelleted DNA was washed with 70 % ethanol and dissolved in sterile water. DNA was quantified on a Qubit 4 fluorometer (Thermo Fisher Scientific, USA).

Total RNA isolation. Colony cells were pelleted at 400g for 5–7 min. The precipitate was resuspended in TRIzol Reagent (Thermo Fisher Scientific, USA). Total RNA was isolated in accordance with the manufacturer’s instructions. RNA content was measured on a Qubit 4 fluorometer (Thermo Fisher Scientific, USA).

Obtaining cDNA. Reverse transcriptase PCR was performed on the poly-A mRNA template using a T100 Thermal Cycler (Bio-Rad Laboratories, USA) and an MMLV RT kit (Evrogen, Russia) according to the manufacturer’s protocol.

Dot blot hybridization. DNA samples isolated from mouse and human colony cells were used to quantify telomeric DNA. DNA samples were sonicated to a size of 100–500 bp. DNA was denatured in 0.2 M NaOH at 100 °C for 10 min, and equal quantities of DNA were applied to the Hybond N membrane using specialized equipment, a dot chamber. The samples were annealed to the membrane for 10 min using an ultraviolet lamp and stored until hybridization.

The membrane with attached DNA was transferred to 50 mL of a pre-hybridization buffer containing 0.1 % SDS, 5×SSC, 5× Denhardt’s solution, and 100 μg/mL yeast total RNA, and incubated at 37 °C for 1–3 h. The labeled DNA sample 54 bp (P32 oligonucleotide G-probe – (TTAGGG)9; C-probe – (CCCTAA)9) was denatured by 10-min boiling and added to 50 mL of the hybridization buffer containing 0.1 % SDS; 5×SSC; 5 % dextran sulfate 500,000; and 100 μg/mL yeast total RNA. The pre-hybridization solution was removed, and the hybridization buffer containing labeled material was added to the membrane after stirring. Hybridization was carried out at 37 °C overnight under constant stirring. After hybridization, the membrane was washed thrice with a solution containing 0.1 % SDS and 0.1×SSC (for 15 min each time) at 37 °C. The hybridization regimen (the buffer system, temperature, and number of washings) of short oligonucleotides was selected empirically in numerous experiments with radioactive isotopes of phosphorus and lies within the range of 37–42 °C (Dolgova et al., 2012).

The membrane with the samples transferred to it was projected onto a K-type screen. Radioisotope-labeled samples were scanned using the PharosFX system. The recorded images were analyzed employing the Quantity One software using the spot density parameter (intensity/mm2).

Pulsed-field gel electrophoresis. Rat colony cells were used for quantifying telomeric DNA by pulsed-field gel electrophoresis. The cells were pooled, washed to remove the methylcellulose medium, and counted in a Goryaev chamber. The colony cells were embedded into blocks based on 1 % low melting point agarose (5×105 cells per block). Before the analysis, the blocks were stored in 0.5 M EDTA at 4 °C. Before pulsed-field gel electrophoresis, the blocks were rinsed in TE buffer and incubated with a lysis buffer (50 mM EDTA, 1 % sarcosyl (Serva, Germany), 1 mg/mL proteinase K (Thermo Fisher Scientific, USA)) for 20 min at 50 °C. Next, the low melting point agarose blocks were fixed in agarose block pockets and subjected to electrophoretic separation in a pulsedfield gel electrophoresis system according to the following regimen: forward – 3 s; reverse – 1 s; RAM-factor – 0.9.

DNA was then transferred to a Hybond N membrane using the capillary method in 20×SSC (Maniatis et al., 1984). DNA samples were attached to the membrane for 10 min using an ultraviolet lamp and stored until hybridization. Hybridization with the P32-labeled oligonucleotide and scanning of radioisotope- labeled samples were then performed in a manner identical to that for dot blot hybridization assay.

Analysis of TERT expression. Bone marrow cells isolated from bone marrow sections from patients with Hodgkin lymphoma were incubated in the presence of inducers (hDNAgr, angiogenin, angiogenin+hDNAgr) and without them (untreated bone marrow cells, control) in IMDM for 1 h in an atmosphere of 5 % CO2, at 95 % humidity and 37 ℃. Next, the bone marrow cells were cultured in methylcellulose medium for 15 days. When isolating cells from the methylcellulose medium, the colonies were pooled and washed to remove the medium according to the manufacturer’s instructions. The colony cells were then counted in a Goryaev chamber and incubated again with inducers. After inducer activation or without it, the cells were re-pelleted for 10 min at 400g, resuspended in DMEM/F-12 (1:1) medium (BioloT, Russia) supplemented with 10 % fetal bovine serum (Capricorn Scientific, Germany), 100 μg/mL gentamicin (Dalkhimpharm, Russia) and 1 μg/mL amphotericin B (Sintez, Russia), and inoculated into wells of a 24-well plate. A sample of cells was collected and divided into two parts consisting of equal amounts of cells 1, 2, 4, 8, 16, and 32 h after re-induction. The zero point corresponded to the colony cells before re-treatment with inducers.

One portion of the cells was pelleted; the precipitate was lysed in TRIzol Reagent, and total RNA was isolated. RNA samples were pooled into two groups: 0–4 and 8–32 hrs. RT- qPCR was performed on the poly-A mRNA template using a T100 Thermal Cycler amplifier and an MMLV RT kit according to the manufacturer’s protocol. qPCR was conducted in 96-well plates using the BioMaster HS-qPCR SYBR (2×) mix according to the manufacturer’s protocol on a QuantStudio 5 Real-Time PCR System (Applied Biosystems, USA). The primer sequences are summarized in the Table. The qPCR analysis of each sample was performed in three replicates. The relative expression level was determined by the 2–ΔΔCt method. The 0–4 h group of samples was used as a control group; the expression level of the target gene in them was taken as unity. Rplp0 was used as the reference gene. The PCR protocol was as follows: 95 °C for 5 min, 40 cycles of 95 °C for 20 s, 57 °C for 30 s, 72 °C for 30 s; the final melting step with slow heating from 60 to 95 °C.

**Table 1.**
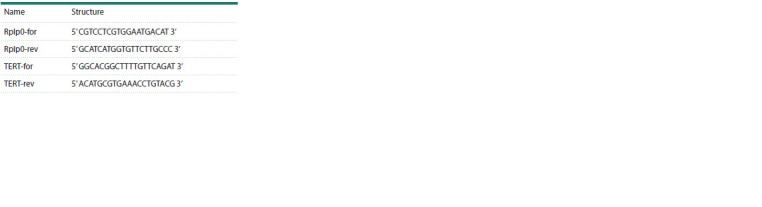
Sequences of the primers used

The other portion of the cells was pelleted and resuspended in saline solution. Protease inhibitors were added to the cell suspension: PMSF, N-ethylmaleimide, and TPSK to a concentration of 1 mM and aprotinin, to a final concentration of 2 μg/mL. A sample buffer (66 mM Tris-HCl, pH = 6.8; 26.3 % glycerol; 2.1 % SDS; and 0.011 % bromophenol blue) was then added; lysates were boiled at 96 °C for 10 min and centrifuged for 5 min at 12,000 rpm. The lysates were used to conduct electrophoresis, and samples were not pooled over time. The samples were equilibrated according to the number of lysed cells before being applied to the electrophoresis system. Commercially available recombinant human TERT protein (Cloud-Clone-Corp, USA) (2 μg per lane) was used as a control. Western blotting with antibodies was performed after electrophoresis and transfer to the nitrocellulose membrane. Non-specific binding was blocked by incubation in 0.01 M phosphate-buffered saline (PBS) supplemented with 0.02 % Tween 20 overnight at 4 °C. Membranes were then incubated with polyclonal antibodies specific to human TERT (Cloud-Clone-Corp, USA) or monoclonal primary antibodies specific to human TERT (Antibody System, France) and antimouse IgG (H+L) secondary antibodies (Affinity Biosciences, USA). Western blotting was performed using an ECL Western blotting detection system (Abcam, UK) and visualized using an iBright imaging system (Thermo Fisher Scientific, USA).

Statistical analysis was carried out using the Statistica 8 software (StatSoft, USA). Statistical significance was assessed using the Mann–Whitney U-test; the differences were considered significant at p < 0.05.

## Results

**Choosing the adequate method for quantifying the telomeric DNA content**

In order to choose the method for quantifying the telomeric DNA, mouse bone marrow cells were treated with hDNAgr and angiogenin activators and inoculated onto methylcellulose. The cells were harvested after nine days. DNA was isolated from a portion of the cells, followed by real-time PCR and dot blot hybridization. Some cells from the same sample were treated with colchicine, and FISH was carried out. Hence, the experiments were conducted using the same cell material at a single time point, “here and now”, so we successfully assessed the adequacy of each approach for quantifying telomeric DNA in the analyzed samples (Supplementary Material 3).

The findings indicated that real-time PCR and FISH used for analyzing telomere length under the selected experimental conditions yielded conflicting results that could have mechanistic interpretation. Only dot blot hybridization allows one to detect high statistically significant difference in changes in telomeric DNA content. Therefore, we chose quantitative dot blot hybridization for measuring telomeric DNA content. This approach allows one to directly quantify the content of DNA homologous to the probe being used in the experimental sample regardless of the circumstances summarized in Supplementary Material 3.

**Quantification of telomeric DNA content in colony cells by dot blot hybridization**

There can be several reasons for the increased telomeric DNA content in HSC progeny that was treated as part of bone marrow cells and gave rise to colonies with a higher telomeric DNA content: 1) integration of telomeric DNA that is initially present in the hDNAgr sample into the HSC genome and its amplification as part of genetically homogeneous colony cells; 2) amplification of cyclic telomeric repeats present in hDNAgr (rolling circle amplification or alternative lengthening of telomeres); 3) induction of endogenous HSC telomerase or a transient telomerase gene incorporated along with extracellular hDNAgr internalized into HSCs, stochastically containing telomerase gene DNA; 4) activation of quiescent HSCs, previously never activated by life events, containing an initially given, maximum possible, number of telomeric repeats (and thus telomeric DNA); 5) the increase in the amount of telomeric DNA is a consequence of the presence of colonies of residual initial hDNAgr in the cells; 6) mixed variants are also possible.

*Mouse and human bone marrow cells*

**Fig. 2.**
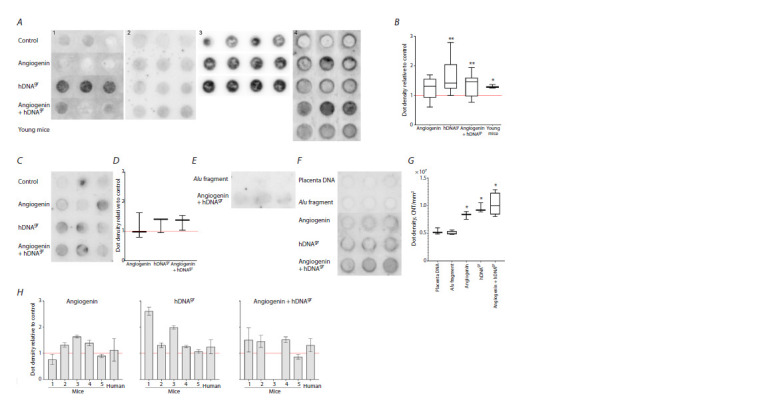
Quantitative dot blot hybridization of DNA extracted from colonies of hematopoietic stem cells within mouse (A, B) and human bone marrow (C–G) without induction (Control) and after induction with angiogenin, hDNAgr, and angiogenin+hDNAgr using a telomeric repeat (54 bp, n = 9) as a probe. A1, A3, C, F – C-probe; A2, A4 – G-probe. DNA of young mice (A4), human placenta DNA (F), and DNA extracted from colonies derived from HSCs within bone marrow cells subjected to treatment with the pBSM13-AluI-pBSM13 PCR fragment (E, F). The membrane was analyzed using a phosphor imaging system. Signal intensity was analyzed in the Quantity1 software. A, C, E, F – images of the membranes after hybridization. B, D, G – the diagrams showing spot density (intensity/mm2) with respect to the control group where spot density was taken as unity (the red line). Significant differences were determined using the Mann–Whitney U-test compared to the control group (B) and compared to the group treated with the AluI fragment (G); * p <0.05, ** p < 0.01. H –comparative analysis of hybridization intensity (the telomeric DNA content) from individual experiments between colony DNA samples after treatment with angiogenin, hDNAgr, or angiogenin+hDNAgr.

The telomeric DNA content in HSC progeny cells treated within bone marrow cells with hDNAgr activators, angiogenin and their combination, in the mouse model and in human bone marrow cells using the quantitative dot blot hybridization was estimated (Fig. 2). Experiments were repeated multiple times (see Figure 2 captions) with DNA from different extractions and using forward and reverse hybridization probe primer. Two approaches to normalizing DNA quantity in the treated cell samples were selected. First, DNA quantities were normalized with respect to intercalator (Qubit), and quantitative dot blot hybridization was performed (mouse model, human bone marrow cells). Second, normalization was performed with respect to the number of colony cells taken into the treatment (rat model).

Figure 2H compares the results of hybridization of angiogenin, hDNAgr, and angiogenin+hDNAgr. One can see that the hybridization intensity varies differently in the angiogenin samples in different experiments. For hDNAgr, the hybridization intensity is always higher than that of the control; telomeric DNA content for the hDNAgr inducer exceeded that in the control 1.1–2.5-fold. When using a combination of two inducers, the hybridization signal was insignificantly higher than that in the control. The findings revealed that the considered trait for angiogenin as a monopreparation is unstable.

Finding an association between colony type and hybridization intensity. We compared the dependence of hybridization signal intensity (the telomeric DNA content in the sample) on the type of colonies in four independent experiments, where the indicated parameters were taken into account. Two lineages, BFU-E and CFU-GM, were analyzed (Supplementary Material 4).

These findings indicated that there are fundamentally different reasons for the increase in quantities of detectable telomeric DNA upon treatment with angiogenin and hDNAgr. For angiogenin, the increase in telomeric DNA content can possibly be related to induction of G0 activity of CFU-GM colonies, which had previously remained inactive in the bone marrow and contained the embryologically predetermined quantity of telomeric DNA. For hDNAgr, comparison of all the data obtained for both model systems revealed no correlation between the hybridization intensity and prevalence of a certain colony type.

Assessment of the intensity of hybridization with P32- labeled telomeric probe using the pBSM13-AluI-pBSM13 PCR fragment as an inducer. Special mention should be given to the results of comparative hybridization with DNA isolated from colonies derived from HSCs within bone marrow cells treated with the pBSM13-AluI-pBSM13 PCR fragment, with placenta DNA and DNA isolated from colonies on day 15 after treatment with inducers. It appeared that the PCR DNA fragment did not stimulate the increase in telomeric DNA content in colony cells (Fig. 2E–G). This fact means that extracellular DNA fragments (in this particular experiment) do not induce endogenous telomerase activity.

Justification of the feasibility of changing the hybridization intensity depending on quantity and composition of internalized DNA fragments, as well as the variant of the P32-labeled telomeric (C/G) probe. It is noteworthy that changes in hybridization intensity in the samples exposed to hDNAgr in different experiments can be related to the quantity of telomeric DNA internalized by the cell. Since the cell may contain approximately 0.2 % (mouse) to 0.02 % of extracellular fragments (humans) (Potter et al., 2024; Ruzanova et al., 2024), the result of competitive internalization will always be vague when it comes to the qualitative composition of the internalized fragments. It means that the number of telomeric repeats can vary significantly from experiment to experiment. Furthermore, the variation in hybridization signal intensity could be because either forward or reverse primer had been used. An analysis of hybridization intensity using two different probes indicated that DNA homologous to the G-tail (C-probe) underwent maximum amplification. DNA homologous to the C-tail (G-probe) was also amplified, but not significantly.

Comparison of the intensity of hybridization response to P32-labeled telomeric DNA probe between the DNA extracted from colonies of the control sample and DNA extracted from the liver of young animals. The intensities of hybridization response to the P32-labeled telomere DNA probe were compared to DNA extracted from colonies of the control sample and from the liver of young animals (Fig. 2A4). One can see an unambiguously interpretable rise in the intensity of hybridization response in the young animal sample. This result supports the known fact that old organisms have a lower telomeric DNA content in HSCs than young individuals. Moreover, this finding indicates that if inducers activated proliferation of quiescent HSCs of embryonic origin, the hybridization pattern would not differ significantly for the samples obtained from young animals and experimental mice.

Assessment of the chances that residual hDNAgr can remain in HSCs after they are treated with this DNA within bone marrow cells, which may cause an artifact of increased telomeric DNA content. If the DNA internalized by the cell is not integrated into the genome, there is a chance that it is present as extrachromosomal material for a long time, which may produce this artifact of increased telomeric DNA content (Dolgova et al., 2012).

The quantity of foreign DNA in the progeny cells of human bone marrow cells on day 15 of cultivation on methylcellulose after HSCs within bone marrow cells had been treated with TAMRA-labeled AluI repeat DNA flanked by pBS sequences with primers M13 was estimated (Supplementary Material 5). The findings indicate that the AluI repeat DNA molecules, which had initially been internalized into HSCs during primary processing of bone marrow cells, are not detected in colony cells. In other words, the increased telomeric DNA content detected in dot blot hybridization experiments cannot result from the presence of residual original DNA in colony cells. In addition, it follows from the experiments that AluI fragments, together with the nonhomologous ends of pBSM13, are not included in the genome and are not amplified by PCR.

To sum up all the findings obtained, the following conclusion can be drawn. For hDNAgr, the increase in hybridization intensity is not associated with the prevalence of a certain colony type; therefore, it is not associated with HSCs, which have previously been inactive throughout the entire life of the organism. There are several variants for activation of the telomerase gene of exogenous origin, direct integration of telomeric DNA into the HSC genome, or emergence of extra telomeric DNA resulting from replication upon treatment of bone marrow cells with an inducer. The variant that DNA fragments initially internalized by HSCs at an amount sufficient to alter the hybridization response intensity can be persisting in the non-integrated state in the cell throughout the entire time of culturing on methylcellulose is ruled out.

Nevertheless, the cumulative result suggests that there is more likely to be true integration of telomeric DNA internalized by the cell into the genome or emergence of replication- related extra telomere DNA.

For angiogenin, the increase in hybridization intensity can be associated with induction of the CFU-GM-derived cells that previously remained inactive throughout the entire life of the organism. Activation of the endogenous telomerase gene is also possible. Both possibilities are indicated by the results of experiments on internalization of angiogenin protein into primitive murine and human hematopoietic stem cells. It has been demonstrated that angiogenin is internalized by primitive murine Sca1 hematopoietic cells and human CD34+ stem cells (Ruzanova et al., 2024). In this case, the telomerase gene can be activated by angiogenin internalized by active HSCs. Integration is not an option, since there is no necessary substrate.

Hence, this analysis has reliably revealed that changes occur in the cells in response to induction of bone marrow cells by DNA preparation, angiogenin, or their combination, affecting the length of telomeric repeats (the telomeric DNA content) in as many cells as are needed to enable imaging of the observed phenomenon.

*Rat bone marrow cells*

**Fig. 3.**
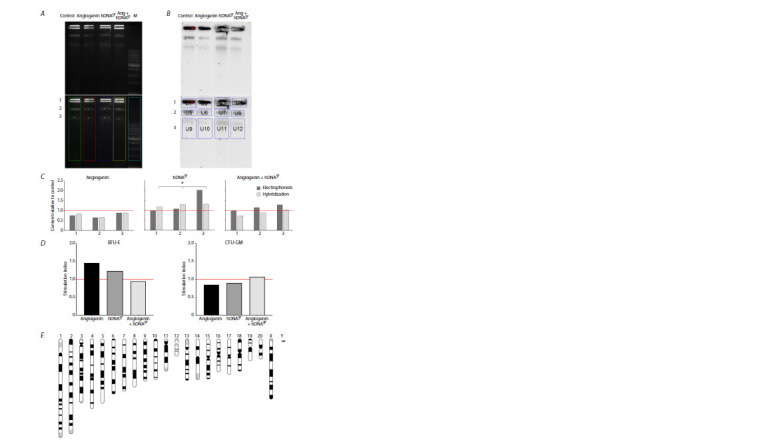
The results of treating rat bone marrow cells with angiogenin, hDNAgr, and angiogenin+hDNAgr. A – electrophoresis with DNA isolated from colonies, from low melting point agarose blocks. In the bottom block, numbers 1–3 denote the fragments used for quantitative analysis. B – hybridization with telomeric repeats (C-probe) of DNA isolated from colonies. In the lower block, numbers 1–3 denote the regions used for quantitative analysis. C – DNA content according to dye luminescence and hybridization intensity for three different fragments compared to the control group (values are taken as unity; shown with a red line). * Differences are significant compared to the control group; p < 0.05, Mann–Whitney U-test. D – the content of the BFU-E and CFU-GM colonies on methylcellulose after treating rat bone marrow cells with different inducers, expressed as an index with respect to the control group (values taken as unity, shown with a red line). E – rat chromosomes (https://rgd.mcw.edu/rgdweb/report/genomeInformation/genomeInformation.html? species = Rat&mapKey = 372&details = true).

For the rat model experiments, the number of colony cells was chosen as the normalization criterion. After being washed to remove methylcellulose, colony cells were embedded into blocks of low melting point agarose (500,000 per block, corresponding to about 3 μg of DNA). The blocks were lysed, and electrophoresis was carried out using a pulse controller as described in the Materials and methods section. The electrophoretic data were analyzed, and Southern blot analysis was conducted. The results obtained are summarized in Fig. 3. The electrophoresis images were processed using the GelPro 3.0 software (Fig. 3A). The relative ratio between DNA quantities in the lanes was estimated from the luminescence of the intercalary dye. In the sample containing DNA-treated cells, DNA quantity in the bands that were subsequently evaluated by hybridization increased by a total of 10 %. Meanwhile, the increase in the three bands was nonuniform: the top two bands almost did not increase, whereas in the third band, the quantity of DNA increased twofold (Fig. 3B).

The hybridization data showed that the number of telomeric repeats in the sample of DNA-treated cells increased by 17–30 % (Fig. 3C, D). The number of telomeric repeats was increased in all the bands, in contrast to the rise in the total amount of DNA. In angiogenin-treated cells, the amount of DNA decreased compared to the control group. It can be related to the fact that a large number of erythroid colonies grew from HSCs treated with angiogenin within the bone marrow (Fig. 3E), which may contain mature red blood cells lacking DNA but which could have been counted during cell selection.

**Analysis of the potential mechanisms for the increase in telomeric DNA content**

Murine HSCs internalize extracellular DNA fragments (Potter et al., 2024). The model system of cryopreserved human bone marrow was used in the main experiments of further studies. It was found that human CD34+ HSCs also capture extracellular DNA fragments. A total of 0.02 % of the haploid genome of extracellular DNA (in a particular experiment) is internalized by the cell (Ruzanova et al., 2024).

Two inducers were selected to analyze the mechanism for the increase in telomeric DNA content: one of those, hDNAgr, carries telomeric DNA as a potential sensing substrate in HSCs, while the other one, angiogenin, does not carry any DNA, including telomeric DNA.

It means that the increase in quantity of detectable telomeric DNA after angiogenin treatment is associated with induction of either endogenous telomerase or activation of quiescent primary HSCs that previously have never been activated and that have been formed and have occupied the bone marrow niches during embryogenesis. Activation and integration of the exogenous telomerase gene are infeasible, since the necessary substrate is lacking.

**Fig. 4.**
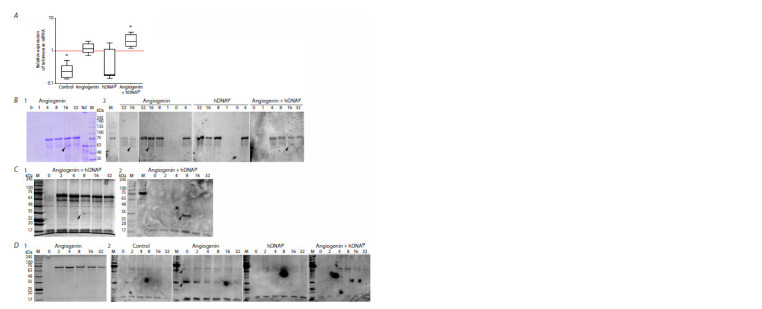
Real-time PCR and Western blotting of RNA and protein lysates to detect telomerase mRNA and telomerase protein. А – real-time PCR of the pooled samples (8–32 h). Values are shown relative to the respective 0–4 h groups (values are taken as unity; indicated with a red line). * Differences are significant compared to the 0–4 h group, p < 0.005, Mann–Whitney U-test. B–D – Western blotting to detect telomerase in the lysates of activator-treated cells; 1 – acrylamide gels with Coomassie staining, 2 – blots with antibodies to telomerase. Incubation time (h) with the corresponding inducers is indicated above the lanes. Arrows indicate specific bands at 63 and 35 kDa. Results from three independent experiments are shown.

The following options are being considered for hDNAgr: the option related to the feasibility of incorporation of hDNAgr per se into the telomeric DNA genome or an increase in the number of G-telomeric repeats as a result of replication repair of the G-strand (tail), which will be observed as a larger quantity of telomeric DNA in the descendants of the primary bone marrow HSCs. hDNAgr can also possibly activate the transient telomerase gene residing within the extracellular fragments internalized into the HSC compartments. The dot blot hybridization data do not support the option that the endogenous telomerase gene in cells accepting the DNA fragment is activated (Fig. 4E–G). An option similar to that described for angiogenin, involving activation of quiescent primary HSCs, which have previously never been activated and which have been formed and have occupied the bone marrow niches during embryogenesis, was not confirmed. Furthermore, the option that the residual amount of hDNAgr is preserved in colony cells after the initial uptake of extracellular fragments by HSCs within bone marrow cells and subsequent contamination of DNA samples in colonies by the residual amount of telomeric DNA, which is initially present in the hDNAgr sample and is sufficient to be sensed by dot blot hybridization, has not been confirmed as well.

The analysis suggested that, at least for angiogenin, the most likely explanation for the increased telomeric DNA content would be induction of telomerase activity by this factor. For hDNAgr, activation of the transient telomerase gene was still possible.

*Assessment of the effect of inducers on telomerase activity*

It was analyzed in direct experiments whether telomerase activity can be activated. The experiments were carried out using the model of HSCs within human bone marrow cells after treatment with hDNAgr, angiogenin, and their combination. It has previously been demonstrated that markers of primitive cells are still retained in colonies grown after HSCs had been activated by the hDNAgr inducer to ~15 % for murine HSCs (c-Kit/Sca-1) and up to 3 % for human HSCs (CD34) (Potter et al., 2024). It meant that reactivation of colony-cultured cells would have similar effects on primitive progenitors and induce similar events in them; in particular, the telomerase gene can be activated in the options suggested above. There would be sufficient material for characterizing cell lysates to detect any telomerase present by real-time PCR or (and) Western blotting.

On incubation day 18, colony cells (pangenomic singlestrand breaks being completely repaired), after activation of HSCs within bone marrow cells by three inducers, were retreated using the same substances. Samples were collected and lysed at the zero point, 1, 2, 4, 8, 16, 32 h after re-induction. Samples were prepared for real-time PCR and Western blotting. For real-time PCR, RNA samples were pooled into two groups, 0–4 and 8–32 h. Time-specific samples were used for Western blotting. The results of the experiments are summarized in Figure 4.

The real-time PCR data (three independent replicates) indicated that when a response develops between 8–32 h after re-induction, telomerase mRNA was synthesized in the samples that had been treated with angiogenin and angiogenin+hDNAgr. In the control sample, telomerase mRNA synthesis was blocked. In the hDNAgr-treated sample, no telomerase mRNA was detected under the selected conditions (Fig. 4A).

Western blotting was conducted using samples corresponding to all the time points. Three independent experiments were performed. Polyclonal antibodies were used in the first one, while monoclonal anti-telomerase antibodies were used in the second and third experiments. The following results were obtained. In the first experiment, when utilizing polyclonal antibodies in the samples treated with angiogenin and angiogenin+hDNAgr, a 63 kDa band corresponding to a cloned fragment (EcoRI-NotI clone 712562) of human telomerase (Cech et al., 1998) was detected 16 h post-induction, which correlates with the overall pattern of telomerase mRNA synthesis (Fig. 4B). Two consecutive experiments utilizing monoclonal antibodies against human telomerase revealed a ~35 kDa band that was undetectable by Coomassie staining (Fig. 4С, D). In the third experiment, a ~63 kDa band was detected along with the 35 kDa band (Fig. 4D). The specific ~35 kDa band was detected in groups of samples treated with angiogenin+hDNAgr (second experiment, Fig. 4С) and angiogenin (third experiment, Fig. 4D). The modes of emergence of this specific band differ for the two experiments. In the second experiment, the ~35 kDa band clearly appeared at the time point of 8 h after starting the re-induction for the angiogenin+hDNAgr sample. No band was detected in the other samples of the second experiment. For the third experiment, an intense ~35 kDa band along with a 63 kDa band was detected at the zero time point (i. e., before the induction in colony cells of the angiogenin-treated sample after washing to remove methylcellulose). As the incubation proceeds, by 32 h of the experiment, the intensity of the 35 kDa band dropped almost to the background value. The 63-kDa band disappeared within the first hour after re-induction. No bands were detected in other images. We found a single publication mentioning a 35-kDa protein related to telomerase activity. During affinity chromatography used for telomerase isolation, the 35-kDa protein was detected along with the 120-kDa and 43-kDa proteins. This protein was not analyzed in this study because it was not detected in preparations of fully purified telomerase (Lingner, Cech, 1996).

The data obtained using two independent approaches indicate that angiogenin activates the molecular mechanisms inducing telomerase activity in HSCs, while exposure to hDNAgr does not abolish the activity of this mechanism.

The results obtained mean that: 1) hDNAgr does not induce expression of the telomerase gene; therefore, the increased telomeric DNA content cannot be related to telomerase activity; 2) angiogenin induces expression of the telomerase gene, and the increase in the telomeric DNA content quantified by dot blot hybridization can be related to this very activity of angiogenin. Exposure to a combination of angiogenin and hDNAgr also enhances synthesis of telomerase mRNA. It was shown in the hybridizations performed that in some cases, the telomeric DNA content in the sample treated with a combination of two inducers is higher than for the samples treated separately. This fact may mean that in this case, the three mechanisms of increasing the telomeric DNA content overlap due to telomerase activity and either direct integration of extra telomeric DNA into the genome of the recipient cell or via replication of quasi t-rings formed by exogenous telomeric repeats during concatamerization and closure into a ring.

## Discussion

The analysis revealed that the two inducers used simultaneously increase telomeric DNA content in colony cells via two independent mechanisms: the conventional telomerasedependent complementary synthesis in the case of angiogenin and the alternative lengthening mechanism for telomeres or true integration of telomeric DNA into the telomeric heterochromatin domain in the case of hDNAgr.

Telomerase is a heterodimer formed by the non-coding RNA template (telomerase RNA component with a size of 400 bp, carrying the basic telomeric sequence complementary to the G- strand) used for de novo synthesis of telomeric DNA sequences and the catalytic subunit of the enzyme (telomerase reverse transcriptase, TERT). The telomerase complex orchestrates telomere length homeostasis by inserting telomeric repeat sequences to the 3ʹ end of the chromosome using the RNA template (Fig. 1B). With a few exceptions (e. g., lymphocytes and endothelial cells), most human somatic cells exhibit no telomerase activity, mostly because of suppression of TERT expression. On the other hand, stem cells, germline cells, and most tumors exhibit it (Chan, Blackburn, 2003; Giraud-Panis et al., 2010; Nandakumar, Cech, 2013; Soman et al., 2022).

As shown previously, angiogenin is internalized into Sca1 (mouse) and CD34 (human) hematopoietic stem cells (Ruzanova et al., 2024). It has also been demonstrated using human bone marrow cells that angiogenin treatment stimulates the GM hematopoietic lineage and induces telomerase activity. Earlier, it was revealed (Goncalves et al., 2016) that recombinant angiogenin stimulates proliferation of myeloid progenitors (like in our experiments), while enhancing the quiescent properties of stem cells. These characteristics are attributed to generation of stress-induced tiRNAs, reduction of the synthetic activity of blood stem cell, enhanced ribosomal RNA synthesis, and stimulation of protein synthesis in myeloid progenitor cells. It is possible that the emergence of telomerase in cells of GM colonies, which has been demonstrated in our study, is a consequence of the first process (stimulation of proliferation of myeloid progenitor cells).

Internalized DNA fragments initiate the formation of nicks required for chromatin rearrangement toward the selected pathway of terminal differentiation of progenitor cells, which actually trigger the mechanism of this differentiation (Ruzanova et al., 2024). A similar concept was discussed in ref. (Sjakste, Riekstiņa, 2021), where it was suggested that chromosomal DNA damage in stem cells can trigger differentiation. Nick-induced chromatin perturbations (chromatin relaxation) will result in an induced recombinogenic situation and activation of the recombination repair machinery comprising numerous active and structural proteins (Nabetani, Ishikawa, 2011). This fact means that the initially extracellular DNA fragments residing in the intranuclear space of HSCs can participate in recombination events that they initiated. The results of the present study suggest that the increase in telomeric DNA content when using hDNAgr as an inducer implies either integration of extracellular fragments carrying telomeric repeats into homologous telomeric regions or activation of the mechanism of alternative lengthening of telomeres using concatamerized cyclic telomeric repeat sequences.

**Fig. 5.**
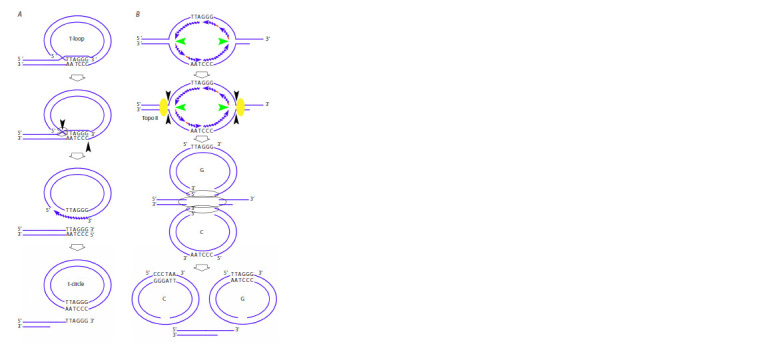
The mechanisms of formation of an extrachromosomal circle. А – formation of t-circles as a result of detachment of the telomere end structure of the t-circle. B – formation of G- and C-circles as a result of replication fork stalling, induction of break-induced repair, and G- and C-strand bulging with involvement of Topo II, the NHEJ mechanism, and DNA-PK activity (Zhang et al., 2017).

An analysis of the intensity of hybridization response revealed that the telomeric DNA content was significantly increased in the analyzed samples, suggesting that the mechanism of alternative lengthening of telomeres was involved in this process to a greater extent. One of the most characteristic features of cells having an active mechanism of alternative lengthening of telomeres is the presence of extrachromosomal telomeric circles, which are either double-stranded (t-circles) or partially single-stranded (C- or G-circles) Cesare, Griffith, 2004; Wang et al., 2004; Henson et al., 2009). The t-loops are closed double-stranded DNA. C-circles are extrachromosomal telomere DNA with a C-strand forming a circle and a broken G-strand annealed to it. G-circles are extrachromosomal telomere DNA, with the G-strand forming a circle and the broken C-strand annealed to it. The origin of these extrachromosomal structures is usually attributed to nick-initiated replication of telomeric DNA (Fig. 5). Break repair is known to be stimulated by induction of telomeric double-strand breaks (McEachern, Haber, 2006; Dilley et al., 2016) and, as suggested by the present study, single-strand breaks (nicks). Break-induced replication can be initiated by integration of the strand end of the ruptured telomeric agglomerate between the strands of the intact telomere and proceed via the branch migration mechanism. The migrating D-loop copies telomeric repeats from the strand invasion point towards the end of the donor telomere (Saini et al., 2013; Wilson et al., 2013), which is accompanied by restoration of telomere length and structure.

**Fig. 6.**
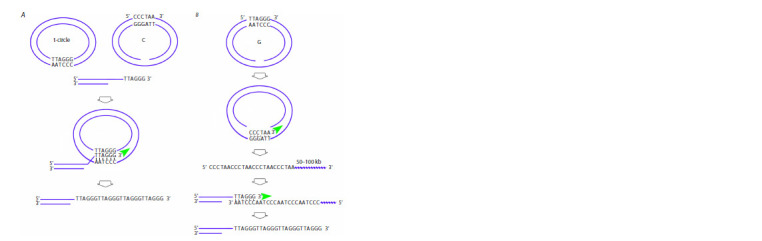
The mechanisms of telomere lengthening. А – lengthening of the telomeric tail at the t-circle and C-circle. B – lengthening of the telomeric tail at the G-circle (see explanation in the text).

Another mechanism of restoring telomere length is associated with insertion of the 3ʹ end of the telomeric G-strand between the strands of extrachromosomal t- or C-circles. Rolling circle replication is induced in this case, leading to accumulation of a single-stranded G-rich telomeric strand (Fig. 6A) (Nabetani, Ishikawa, 2011; Lu W. et al., 2013; Doksani, 2019).

Two mechanisms of formation of t-, C-, and G-circles have been described. t-circles can be formed by intrachromosomal recombination and t-loop liberation as a result of Holliday recombination of the 3ʹ end of the t-loop annealed to the complementary sequence of the 3ʹ–5ʹ strand (Fig. 5A) (Wang et al., 2004; Nabetani, Ishikawa, 2011; Claussin, Chang, 2015; Doksani, 2019; Jones et al., 2023). In the other case, both variants of the C- and G-circles are formed (Fig. 5B).

It was demonstrated that both variants of DNA circles containing telomeric repeats are bulged upon replicative stress associated with replication fork stalling in difficult-to-replicate telomeric heterochromatin or with chromosomal DNA damage (e. g., double-strand breaks or nicks). In this case, the G-circle can initiate the rolling circle replication starting with the nick and synthesis of the C-rich tail carrying telomeric repeats of the 3ʹ–5ʹ strand up to 100 kbp long, which can be detected both using a phi29 polymerase kit and in vivo (Zhang et al., 2017). The amplified single-stranded C-rich tail can assumedly be annealed to the truncated G-telomeric strand (e. g., after a t-loop is separated from the telomere because of replicative stress) and as a homologous template to stimulate synthesis of the truncated G-rich strand (Zhang et al., 2017). Topo II, the NHEJ mechanism, and DNA-PK activity are involved in circle formation in this case (Fig. 6B).

The more than twofold increase in telomeric DNA content in some experiments is attributed to telomeric repeat amplification via the putative rolling circle replication mechanism using circular structures as a template and nick-initiated replication. For this type of amplification, the single-stranded G-strand region can be as long as 70–100 kbp (Doksani, 2019; Jones et al., 2023) (Fig. 6A, B).

The large number of C-circles in the cell is the basic criterion for the mechanism of alternative lengthening of telomeres. The single-stranded 3ʹ end of the 5ʹ–3ʹ G-strand can be paired with both the t- and C-circle to form a D-loop (Fig. 6A). The following multiple rounds of rolling circle replication amplify telomeric repeats. The number of telomeric repeats synthesized via this mechanism can be degenerate and will differ for different telomeres in different chromosomes (Lee et al., 2014; Jones et al., 2023).

When extracellular fragments carrying telomeric repeats are internalized into the nucleus, the following events occur. A recombinogenic situation triggered by the emergence of single-strand breaks is initiated. If the factors activated by the nick-initiated recombinogenic situation are similar to those activated by the recombinogenic situation initiated by double-stranded breaks (Dolgova et al., 2013), the internalized double-stranded fragments will promptly form a circle (Dolgova et al., 2013; Potter et al., 2018, 2024). During the time they exist in a linear form, they can be integrated into the genome via the ends in/ends out mechanism. Once ligated into a circle, these structures will be virtually indistinguishable from the t- and C-circles formed via the mechanism of alternative lengthening of telomeres. This means that the amplification of telomeric DNA during the internalization of extracellular DNA in HSCs is mostly associated with the elongation of the G-chain of the telomere as a result of activation of replicative synthesis by the rolling ring mechanism, presumably induced by nicks. The significant (more than twofold) increase in telomeric DNA content in some experiments can be attributed to this very fact.

**Fig. 7.**
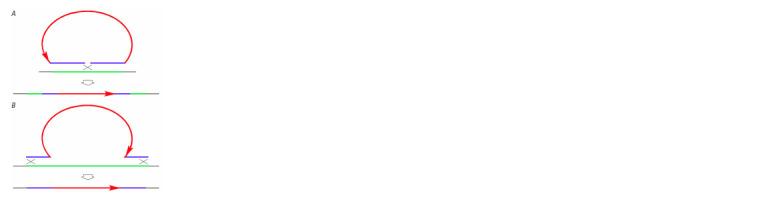
The mechanisms of ends in/ends out integration of extracellular DNA fragments into the recipient genome. А – ends in integration. В – ends out integration.

Integration of extrachromosomal fragments carrying telomeric repeats (leading to extension of telomeric DNA) can also be implemented via the ends in/ends out homologous recombination mechanism (Fig. 7) (Rubnitz, Subramani, 1984; Hastings et al., 1993; Cromie et al., 2001; Li et al., 2001; Langston, Symington, 2004). Other mechanisms of homologous recombination (single strand annealing or gene conversion) will not increase telomeric DNA content.

## Conclusion

Hence, the conducted studies demonstrate that extracellular DNA fragments internalized by HSCs and carrying telomeric repeats can be either directly integrated into telomeric heterochromatin or become a template for alternative lengthening of telomeres, which is accompanied by a rise in telomeric DNA quantity and, presumably, an increase in telomere length.

## Conflict of interest

The authors declare no conflict of interest.
